# Feasibility study of emergency intervention for vascular injury outside the hospital

**DOI:** 10.1186/s40779-016-0106-1

**Published:** 2016-11-29

**Authors:** Ming Liang, Jing-Jing Rong, Jing-Yang Sun, Xiao-Zeng Wang, Fei Li, Geng Wang, Yan-Chun Liang, Ya-Ling Han

**Affiliations:** Department of Cardiology, General Hospital of Shenyang Military Region, Shenyang, 110016 China

**Keywords:** Vascular injury, Pre-hospital emergency, Intervention, Cabin

## Abstract

**Background:**

Minimally invasive surgery in the field of traumatic vascular injury diagnosis and treatment has achieved good results. This study was designed to determine whether pre-hospital emergency intervention is feasible for vascular injury in a field intervention cabin under the condition of war or a disaster site.

**Methods:**

Different types of animal experiments of vascular injury intervention were performed in a field intervention cabin. Treatment capacity was evaluated by data collection, including duration of surgery, clinical evaluation, image clarity, and equipment handling. Environmental adaptability and mobility were evaluated by maneuverability and long-distance mobility.

**Results:**

A total of 56 surgeries (7 types) were performed in the field intervention cabin. Digital subtraction angiography (DSA) had good imaging performance. A total of 4800 km of long-distance mobility was performed, and all the equipment operated normally without any equipment failure. We participated in the medical service maneuver twice. The cabin unfolded and worked properly. There was no equipment damage during the medical service maneuver.

**Conclusions:**

Use of a field intervention cabin under the conditions of war or disaster is feasible for pre-hospital emergency intervention of vascular injury.

## Background

War and natural disasters are often accompanied by heavy casualties. Among different injuries, vascular injury is one of the major causes of death and disability. Timely and effective pre-hospital emergency care can significantly reduce the fatality rate, decrease the incidence of disability and improve the prognosis [1, 2]. Furthermore, minimally invasive surgery in the field of traumatic vascular injury diagnosis and treatment has achieved good results [3–5]. However, due to the limitations of inner space, short power supply, large equipment size, and inconvenient transportation, minimally invasive surgery is only used in a hospital in a fixed catheterization lab and rarely used in pre-hospital emergencies. To date, no reports on the use of a mobile catheterization lab at a warfront or disaster site have been offered [6, 7]. Thus, the purpose of the present study was to design a field intervention cabin. The field intervention cabin developed by our team has many advantages, including mobility, clear imaging, and stable performance, which makes pre-hospital intervention of vascular injury at a disaster site possible. The field intervention cabin could significantly increase the ability to address pre-hospital emergencies [8].

## Methods

### Cabin components

The equipment included a bilateral extension cabin (6050 mm × 6260 mm × 2438 mm). The weight was 19.8 t. The cabin included diesel generator power systems, lighting systems, an air purification system, a heating system, an oxygen supply system, water supply systems and other auxiliary functions. Several minimally invasive interventional devices were installed in the cabin, including an interventional angiography system, operating tables, a defibrillator, an invasive blood pressure monitor, a high-pressure injector, X-ray protective clothing, and an image acquisition system. The field intervention cabin was loaded by an off-road truck that could automatically load and unload. The cabin could also be loaded and transported by flat railway cars, ships and cargo aircraft.

### Experimental animals and apparatus

The non-survival procedures were performed under an approved Animal Use Protocol in compliance with regulations and animal care standards. All animals received humane care in accordance with the guidelines published by the National Society for Medical Research (Principles of Laboratory Animal Care) and the National Institutes of Health (NIH, Guide for the Care and Use of Laboratory Animals, NIH publication No. 85-23, revised 1985). Chinese experimental swine (50–70 kg, supplied by the Department of Experimental Animals, Shenyang Northern Hospital) were used in the study. The right femoral artery was accessed surgically, and a 6Fr sheath was initially placed. Intervention apparatuses, including arterial sheaths, venous sheaths, artery covered stents, arterial catheters, and arterial occlusion balloons, were purchased from Johnson & Johnson, USA. The experimental procedures and animal care were approved by the Institutional Animal Care and Use Committee of the General Hospital of Shenyang Military Region.

### Assessment of X-ray radiation doses

To compare the difference of patient radiation doses between the field intervention cabin and a cardiac catheterization lab, a simulation was performed on a radiation simulation irradiated human model (standard plexiglass 200 mm × 200 mm × 30 mm Model VD0203510, IBA Dosimetry GmbH, Germany) [9, 10]. The radiation simulation irradiated human model was exposed on all available image intensifier sizes as well as with a clinically suitable collimation. Dose-area product (DAP) values were recorded by the radiation dosimeters (KermaX® plus, Model 120-131 HS OEM, IBA Dosimetry GmbH, Germany). The entrance dose values were converted to entrance skin dose (ESD) using a backscatter factor of 1.4 based on the used fold sizes and radiation qualities.

### Types of intervention procedures and image quality assessment

Seven types of intervention procedures, including selective hepatic artery embolization, splenic artery embolization, ruptured abdominal aortic treated by covered stent, super-selective renal artery embolization in renal injuries, hemostasis balloon technique in carotid artery rupture, coronary artery angiography and coronary stent implantation, were performed in the present study. Duration of operations and effects of surgery were recorded and analyzed. Fluoroscopy current was 8 to 15 mA. Digital acquisition current was 15 to 35 mA. The longest exposure time was 45 s.

Every coronary artery angiography was obtained from 9 different shooting angles: anteroposterior (AP), cranial (CRA) 30°, caudal (CAU) 30°, left anterior oblique (LAO) 45°, LAO45° + CAR30°, LAO45° + CAU30°, right anterior oblique (RAO) 30°, RAO30° + CRA30°, and RAO30° + CAU30°. To compare image quality between the field intervention cabin and the cardiac catheterization lab, six experimental animals were operated on first in the field intervention cabin and then in the cardiac catheterization lab. Image quality was assessed in a double-blind manner by two experienced diagnostic imaging doctors. Clinical assessment scores of image quality were divided into 3 grades: Grade I: images clearly presented, contrast is good, no artifacts; Grade II: images clearly presented, contrast is not very good, some artifacts, does not affect the diagnosis and treatment; Grade III: images are not clear, contrast is not very good, obvious artifacts, cannot be used for diagnosis and treatment.

### Test of stability and mobility

The stability and mobility of the field intervention cabin were studied in long-distance operation. The ability to adapt to different road conditions and the working status of equipment in the cabin were recorded. Surgeries were performed under different environmental conditions. The working status of equipment, duration of cabin and equipment preparation were recorded and analyzed.

## Results

### Global assessment

**Fig. 1:**
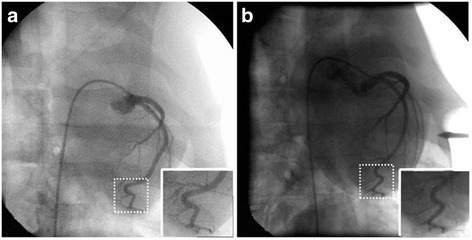
Image comparison. **a** The porcine coronary artery image in the field intervention cabin; **b** The same show of porcine coronary artery in cardiac catheterization lab

From Nov. 2011 to Dec. 2015, a total of 56 experimental animals were subjected to 7 types of interventional procedures (Table 1). Imaging quality suggested that contrast, sensitivity and resolution met the operation requirements (Fig. 1).

**Table 1:** Types of surgery

Procedure	Animals	Counts	Vessel	Quality of image	Duration of surgery (min)	Complication
Coronary artery angiography	Bama mini pig	19	Right femoral artery	I	37	None
Coronary artery stenting	Bama mini pig	11	Right femoral artery	I	37	None
Carotid artery balloon occlusion	Bama mini pig	5	Right femoral artery	I	37	None
Splenic artery embolization	Bama mini pig	6	Right femoral artery	I	39	None
Selective hepatic artery embolization	Bama mini pig	6	Right femoral artery	I	48	None
Abdominal aortic covered stent implantation	Bama mini pig	5	Right femoral artery	I	40	None
Selective renal artery embolization	Bama mini pig	4	Right femoral artery	I	31	None

### Environment adaptability assessment

The field intervention cabin was unfolded in different conditions 23 times. The average unfolding time was 37 ± 11 min. Environment temperatures ranged from −27 to 30 °C. The indoor temperature was 24 °C, which was regulated by the indoor air-conditioning system.

### Mobility assessment

**Fig. 2:**
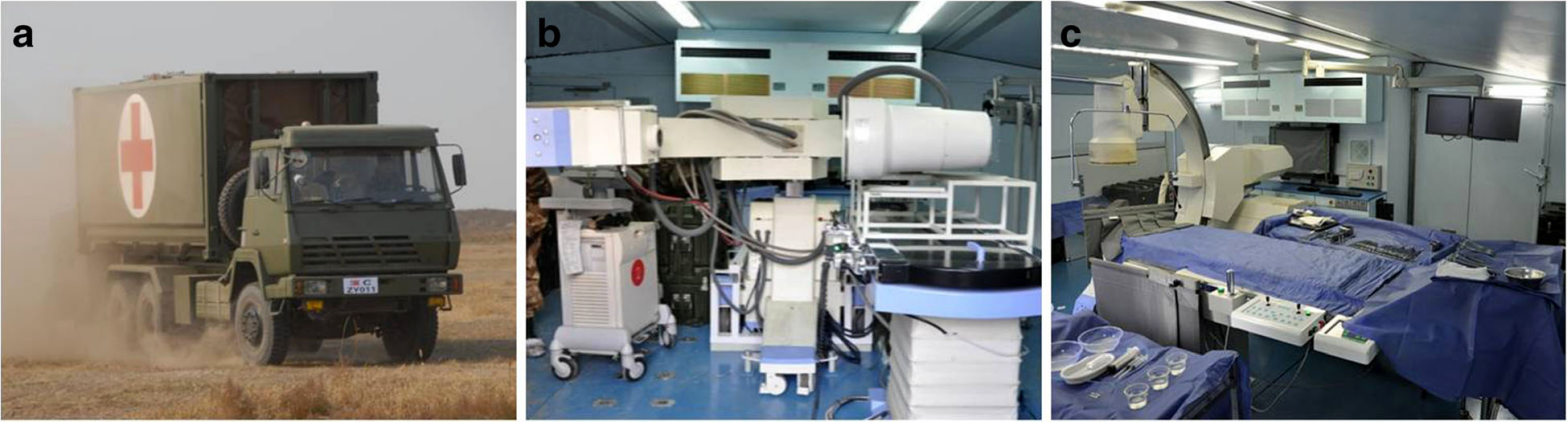
The folded and unfolded states of field intervention cabin. **a** Transporting the field intervention cabin; **b** The interiors of folded field intervention cabin; **c** Working state of the unfolded field intervention cabin

The field intervention cabin was driven a total distance of approximately 12,800 km. The maximum speed was 90 km/h, and the average speed was 70 km/h. The cabin could be driven on city roads, highways, country dirt roads, mountain roads, soft surface roads, and snowy roads. The maximum car ramp slope for the cabin was 40°. No mechanical failures occurred in the cabin (Fig. 2).

### Radiation dose assessment

**Fig. 3:**
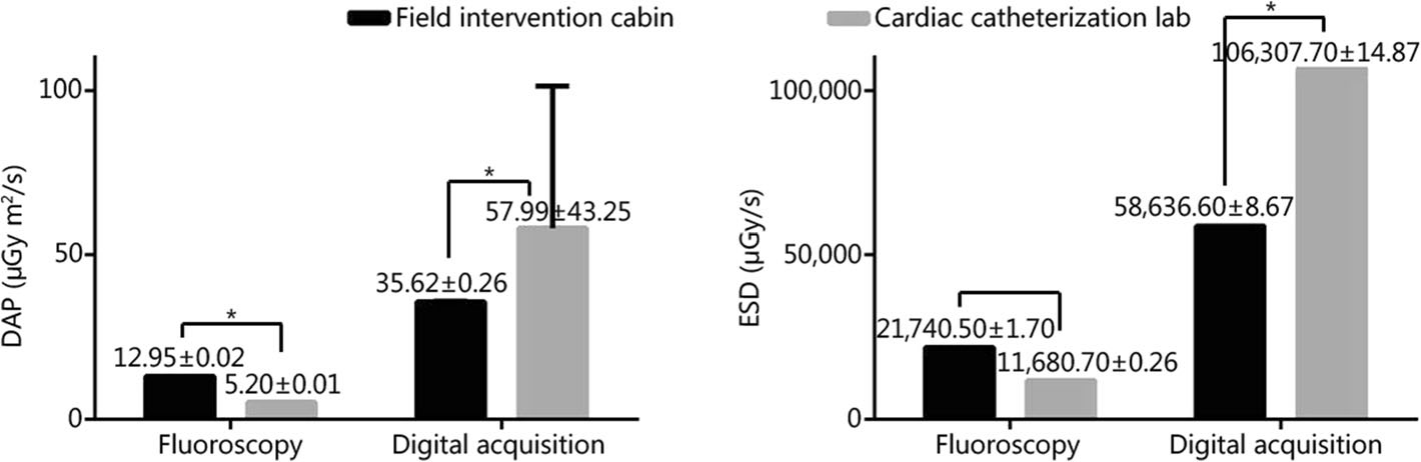
Dose–area product and entrance surface dose for the C-arm and angiographic equipment with an Alderson phantom. The values for digital acquisition account for 10 images plus test exposure. ^*^ *P* < 0.01. The tube potential of fluoroscopy was 80 kV. The tube potential of digital acquisition was 70 kV in field intervention cabin and 72 kV in cardiac catheterization lab. *DAP* Dose-area product, *ESD* Entrance surface dose

Tube potential, DAP, and ESD values of both the field intervention cabin and the cardiac catheterization lab are presented in Fig. 3. Although we identified an upward trend of DAP and ESD when fluoroscopy was performed in the field intervention cabin in a digital mode (DAP, 12.95 ± 0.02 μGy · m^2^/s *vs* 5.20 ± 0.01 μGy · m^2^/s, *n* = 20, *t* = 1552, *P* < 0.0001; ESD, 21,740.50 ± 1.70 μGy/s *vs* 11,680.70 ± 0.26 μGy/s, *n* = 20, *t* = 26,160, *P* < 0.0001), DAP and ESD were reduced in the field intervention cabin under the same output voltage (DAP, 35.62 ± 0.26 μGy · m^2^/s *vs* 57.99 ± 43.25 μGy · m^2^/s, *n* = 20, *t* = 2.313, *P* = 0.0262; ESD, 58,636.60 ± 8.67 μGy/s *vs* 106,307.70 ± 14.87 μGy/s, *n* = 20, *t* = 12,386, *P* < 0.0001).

### Image quality assessment

Angiographic results revealed a qualified image in the field intervention cabin, with the exception of the left shoulder and spleen. Clear images of blood vessels and a good contrast ratio were obtained in the field intervention cabin. Although there was some noise in the enlarged image obtained from the field intervention cabin (Fig. 1), the image quality was similar to that obtained in the cardiac catheterization lab (Field intervention cabin *vs* Cardiac catheterization lab, Rank sum test, *Z* = −0.813, *P* = 0.416, Table 2).

**Table 2:** Image quality assessment

Quality level	Field intervention cabin		Cardiac catheterization lab	
	Counts (*n* = 56)	Ratio (%)	Counts (*n* = 55)	Ratio (%)
I	52	92.9	53	96.4
II	4	7.1	2	3.6
III	0	0	0	0

### Interventional procedures

**Fig. 4:**
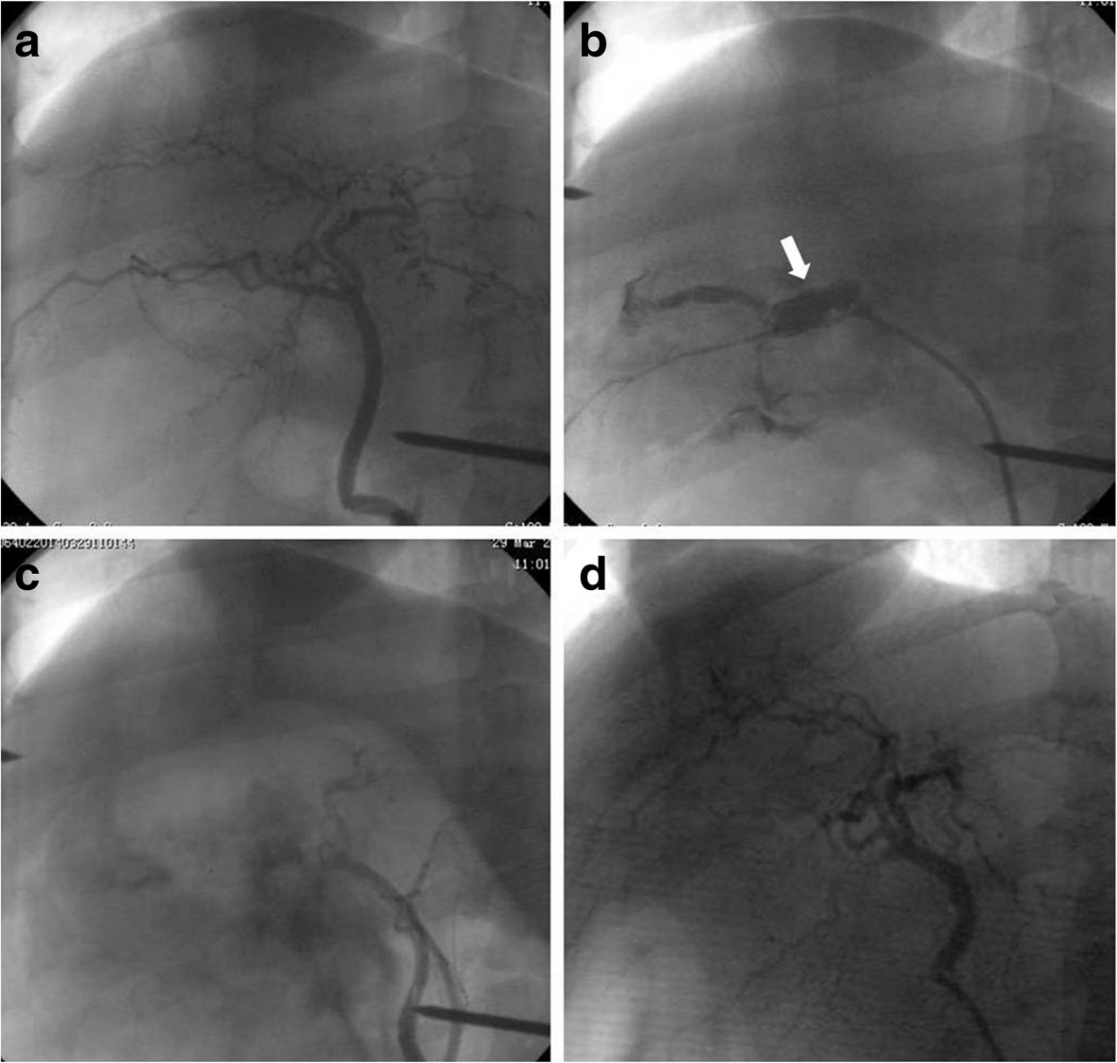
The embolization and hemostasis of injured hepatic artery. **a** Angiographic image of normal liver; **b** Hepatic injury model (*white arrow* shows the retention of contrast agent); **c** Successful embolization of hepatic artery; **d** Angiography 3 weeks after embolization

**Fig. 5:**
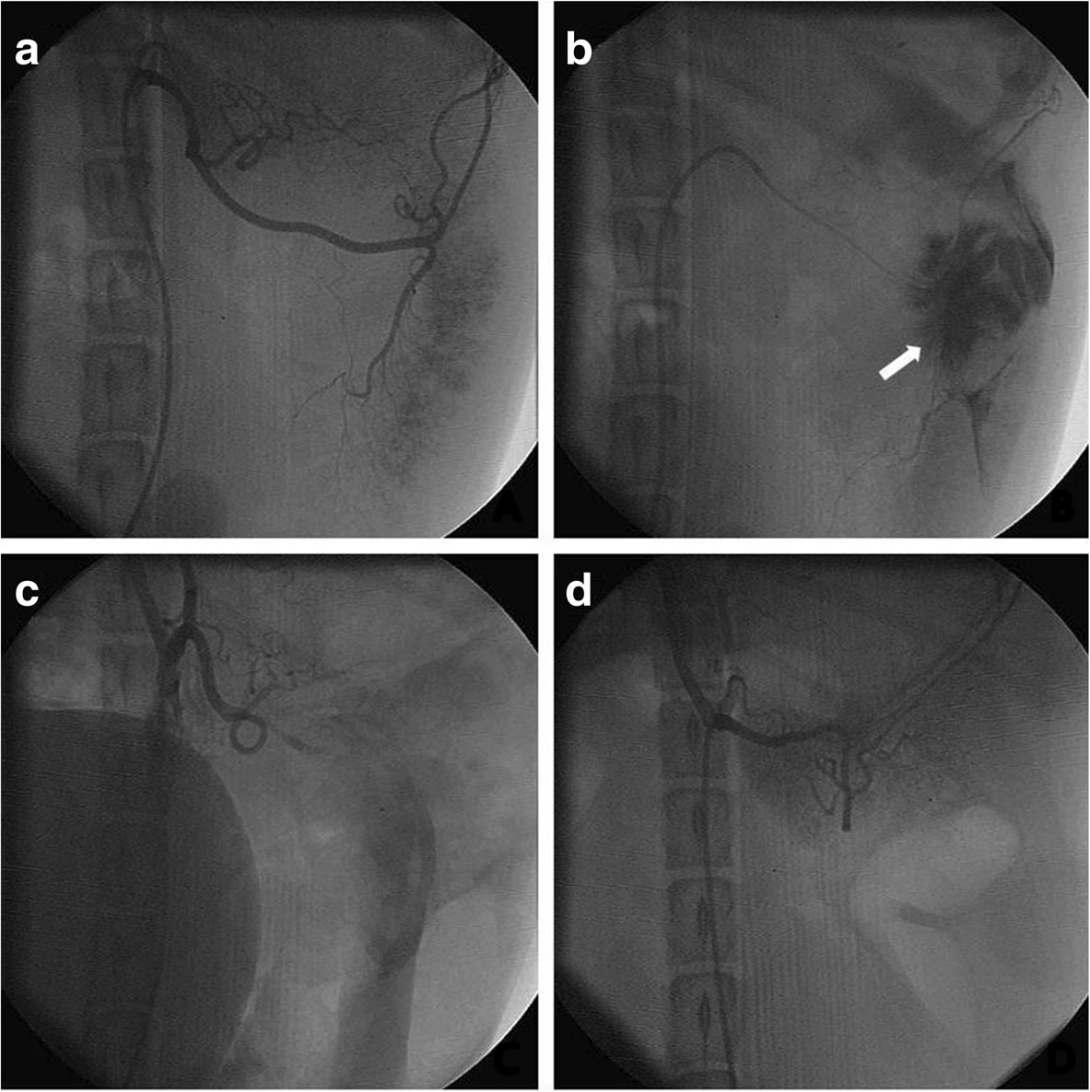
The embolization and hemostasis of injured splenic artery. **a** Angiographic image of normal splenic artery; **b** Spleen injury model (*white arrow* indicates the retention of contrast agent); **c** Successful embolization of splenic artery; **d** 4 weeks after embolization

From 2011 to 2015, 56 Chinese experimental miniature swine were used, and 7 types of emergency interventional procedures, including coronary artery angiography, coronary artery stenting, internal iliac artery balloon occlusion, splenic artery embolization, selective hepatic artery embolization, endovascular stent-graft exclusion, and selective renal artery embolization were performed (Table 1). Imaging data revealed that all of the emergency interventional procedures could be performed in the field intervention cabin (Figs. 4 and 5). The fluoroscopy current was 8 to 15 mA. The digital current was 15 to 30 mA. The maximum exposure time was 45 s. There were no equipment failures. All equipment exhibited good operability in the field intervention cabin.

## Discussion

Data from the present study revealed that the field intervention cabin developed by our team has numerous advantages, such as being small and mobile, provides clear images and stable performance, rapid loading and unloading, and lower emissivity. Compared with data in the cardiac catheterization lab, the field intervention cabin had relatively low DAP and ESD for the C-arm and angiographic equipment with an Alderson phantom but approximately the same image quality, making pre-hospital intervention of vascular injury at a war front or disaster site possible.

Minimally invasive treatment systems that have high mobility and adaptability to the environment are needed at a war front or natural disaster site. The design concept of minimally invasive treatment cabins feature miniaturization of ordinary catheter operating room equipment for truck loading. The cabin was mainly equipped with a medium-sized mobile C-arm X-ray machine, in which interventional treatment for common cardiac and vascular injuries was available. Compared with the existing mobile catheterization lab in the USA, the present field intervention cabin has reduced size and weight, increased mobility and broader environment adaptability, thus facilitating air and rail transport.

The battlefield treatment experience confirmed that vascular intervention can be effectively used in war wound treatment. The present field intervention cabin could meet the demand from the battlefield and disaster sites. Vascular surgery or minimally invasive intervention may be used in the treatment of vascular injury. Minimally invasive treatment was not only capable of blocking blood vessels breaks but also effectively restored blood flow to damaged blood vessels. Furthermore, minimally invasive intervention has numerous advantages, such as reduced trauma and increased safety. Thus, the technique has been widely accepted by clinicians [11, 12]. Increasing attention has been given to minimally invasive technologies in the field of vascular war injury treatment. After carefully analyzing vascular injury data from the US Army in Iraq and Afghanistan, Fox et al. [13] believed that minimally invasive technology reduced the rate of misdiagnosis, crippling, and death. Rasmussen et al. [6] reported that 488 US soldiers suffered from vascular injury during the war with Iraq. In addition, 28% (139 soldiers) received a total of 150 interventional examinations, which confirmed the safety and efficacy of minimally invasive treatment at a war front site. However, because few hospitals have mobile catheterization labs, the minimally invasive technology could only be used in the hospital. Thus, the present field intervention cabin have provided a new method to address pre-hospital emergencies.

The image quality met intervention requirements, whereas the radiation dose was relatively low. Image quality plays a vital role in imaging diagnosis and treatment. We compared the magnified coronary angiography images of animals in the same group using the cabin and a special cardiovascular angiography machine, and found that the cabin provided a relatively poor image quality. However, the cabin revealed no difference when regarding diagnosis and treatment process. Studies revealed an increased cancer risk and skin damage caused by exposure to excessive ionizing radiation; thus, it is very important for both the patients and doctors to be aware of the output radiation dose of the equipment. The total radiation dose is generally attributed to fluoroscopy when we performed blood vessel intervention, minimally invasive treatment and conventional surgery to the common vascular injuries. Results from a standard phantom detection revealed that on the premise that the image quality had the capacity to meet the need of the diagnosis and treatment, the mean radiation dose in cardiac catheterization lab was increased approximately 1.5-fold compared with the field intervention cabin fluoroscopy. Therefore, the field intervention cabin radiation dose was relatively low compared with the cardiac catheterization lab, which is applicable to the diagnosis and treatment of cardiovascular patients.

Animal experiments previously confirmed the cabin’s capabilities to meet war front or disaster treatment needs. To evaluate the working performance of the cabin equipment and their treatment capabilities for vascular injuries, we performed more than 50 surgeries on animal models, including endovascular stent-graft exclusion and interventional embolization hemostatic surgery to hepatic or splenic artery rupture and kidney laceration animal models, which tentatively confirmed that the image quality and equipment maneuverability of this treatment system, as well as the inner cabin environment, can meet the interventional treatment needs of important viscera vascular injury and peripheral vascular injury.

The cabin’s mobility and its environment adaptability enable first aid in catastrophic circumstances. War and natural disasters are often sudden and occur in areas with regional uncertainty [14]. To fulfill an effective treatment to the wounded in the “gold period”, medical service agencies must offer rapid response capabilities and mobile support capabilities [15, 16]. We developed a minimally invasive intervention treatment system with high mobility that can complete emergency first aid in the form of an on-vehicle cabin. With complete consideration of the practical situation of emergency first aid, medical support, and rapid mobility at wartime during the design, the cabin achieves its land transportation via MZX98-12 overall self-loading and discharging special cross-country lorries with stable structure and easy operability. When separated from the lorry, the cabin can be transported by railway flatbed cars, sea or air. First aid and remote mobility drills confirmed the system’s time-saving and effort-saving extension, with great topographical and geomorphological adaptability, mobility and crypticity, thus making it available in various special environments to perform first aid.

## Conclusion

The field intervention cabin makes it feasible to offer pre-hospital emergency intervention for vascular injuries under conditions of war or at a disaster site.

## Abbreviations

AP: Anteroposterior
CAU: Caudal
CRA: Cranial
DAP: Dose-area product
DSA: Digital subtraction angiography
ESD: Entrance skin dose
LAO: Left anterior oblique
NIH: National Institutes of Health
RAO: Right anterior oblique
